# 

**Published:** 1861-01

**Authors:** 


CONTENTS

OF THE

### Chicago Medical Journal for January, 1861.

ORIGINAL COMMUNICATIONS.

PAGE.

Resection of the Knee Joint. By J. W. Freer, M. D., Professor of Surgical and Microscopical Anatomy in Rush Medical College.............. 1

Tendency to Vacuum in the Cerebro-Spinal Cavity. By Daniel Brainard, M. D., Professor of Surgery in Rush Medical College................ 4

Constipation. By J. C. Batford, M. D.................................. 15

SELECTED.

Stimulation *vs.* Depletion. By Patrick Fraser, M. D., Physician to the London Hospital....................................................... 18

Muscular Poisons. By M. Claude Bernard................................ 34

Treatment of Scrofulous Lymphatic Glands. By P. C. Price, Esq., etc.... 38

Morbid Appearances in Death by Cold. By Francis Ogston, M. D., of Aberdeen ....................................................... 43

Proceedings of Societies............................................ 45

EDITORIAL.

Editorial Items...................................................... 49

BIBLIOGRAPHICAL NOTICES.

Books and Pamphlets Received ... .................. .............. 56

To Business Men, etc............................................... 63

Rush Medical College................................................ 64

